# Supplementary material for: New loci and neuronal pathways for resilience to heat stress in cattle
Source: Sci Rep. 2021 Aug 17;11:16619. doi: 10.1038/s41598-021-95816-8 (PMC8371109; doi:10.1038/s41598-021-95816-8)
Supplement: Supplementary file 1 — Supplementary Figures. [file 41598_2021_95816_MOESM1_ESM.docx]

# New loci and neuronal pathways for resilience to heat stress in cattle

**Evans K. Cheruiyot^1,2^, Mekonnen Haile-Mariam^2*^, Benjamin G. Cocks^1,2^, Iona M. MacLeod^2^, Ruidong Xiang^2,3^, Jennie E. Pryce^1,2^**

^1^School of Applied Systems Biology, La Trobe University, Bundoora, Victoria 3083, Australia

^2^Agriculture Victoria Research, AgriBio, Centre for AgriBiosciences, Bundoora, Victoria 3083, Australia

^3^Faculty of Veterinary & Agricultural Science, The University of Melbourne, Parkville 3052, Victoria, Australia

*Corresponding author

E-mail: [Mekonnen.HaileMariam@agriculture.vic.gov.au](mailto:Mekonnen.HaileMariam@agriculture.vic.gov.au)

## List of Supplementary Figure Captions

**Figure S1.** Manhattan plot of GWAS p-values for 29,107 Holstein cows based on 50k SNP set (left panel) and whole-genome sequence variants (right panel; N = 15,098,486 SNPs) for: heat tolerance milk (HTMYslope; A), fat (HTFYslope; B) and protein (HTPYslope; C) yield slope traits. Dashed horizontal lines represent GWAS cut-off of p < 1E-05.

**Figure S2. Manhattan plot of GWAS p-values for 29,107 Holstein cows obtained from 15 million imputed-WGS for**heat tolerance milk (HTMYslope; A), fat (HTFYslope; B), and protein (HTPYslope; C) yield slope traits. The highlighted red points are lead SNPs (most significant) identified using 5 Mb non-overlapping windows at p < 1E-05 (horizontal dashed line).

**Figure S3.** **Overlap of candidate QTLs (p < 1E-05) from single-trait GWAS for heat tolerance milk (HTMYslope), fat (HTFYslope), and protein (HTPYslope) yield slope traits.** QTLs were defined as overlapping if the lead SNPs (most significant) within QTLs are close (within 1 Mb). The two QTLs which overlapped across the 3 slope traits are located around DGAT1 and MGST1 genes.

**Figure S4.** Distribution of linkage disequilibrium (LD) scores between lead SNPs (most significant) for slope traits and nearby (within 1 Mb) lead SNPs for intercept traits identified from single-trait GWAS and multi-trait meta-analyses.

**Figure S5.** GWAS p-values on chromosome 20 at 30 to 36 Mb for heat tolerance milk yield slope (HTMYslope) trait. The left plot (GWAS p-values before conditional analysis), middle (after conditioning slope on the lead SNP defined as the most significant SNP selected from first-round of GWAS; Chr20:32103408), and right plot (GWAS p-values after conditioning with milk yield intercept trait).

**Figure S6.** Conditional GWAS results for heat tolerance milk (HTMYslope), fat (HTFYslope), and protein (HTPYslope) yield slope traits. GWAS for A, B and C were conditioned on milk, fat, and protein yield intercept traits, respectively. The highlighted red points represent the lead SNPs (most significant) within 5 Mb non-overlapping windows across the chromosome.

**Figure S7.** Manhattan plot of p-values obtained from combining conditional single-trait GWAS results for slope traits in the multi-trait meta-analysis. The dashed line is the significant GWAS cut-off at p < 1E-05, while the red circles are the lead SNPs (most significant per QTL).

**Figure S8.** QTL discovery for heat tolerance protein yield slope (HTPYslope) trait around the CALCR gene region in bovine chromosome 4.

**Figure S9.** QTL discovery for heat tolerance protein yield slope (HTPYslope) trait and meta-analysis of slope traits (Meta-HTslope) around the GHR gene region in bovine chromosome 20.


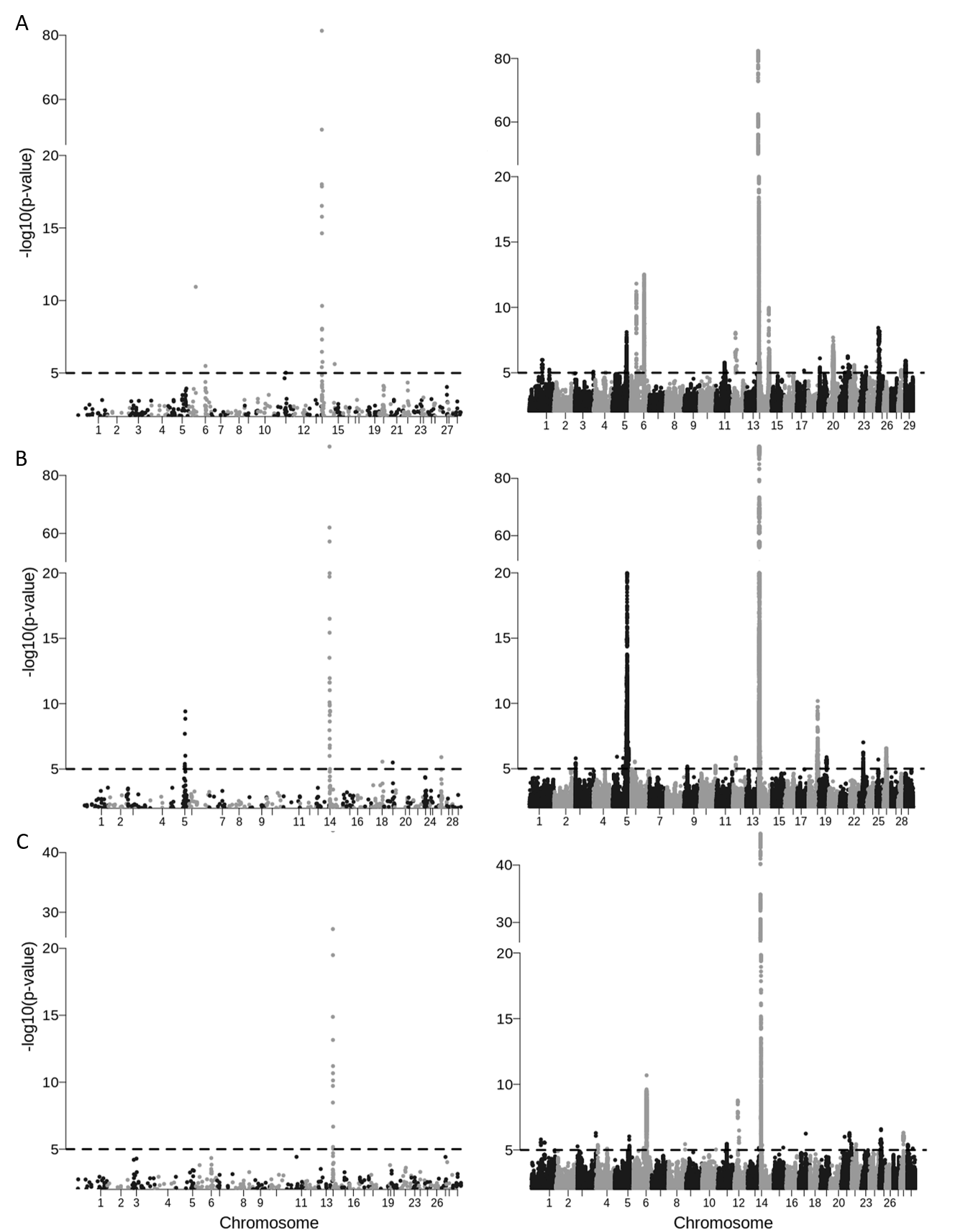


Figure S1 Manhattan plot of GWAS p-values for 29,107 Holstein cows based on 50k SNP set (left panel) and whole-genome sequence variants (right panel; N = 15,098,486 SNPs) for: heat tolerance milk (HTMYslope; A), fat (HTFYslope; B) and protein (HTPYslope; C) yield slope traits. Dashed horizontal lines represent GWAS cut-off of p < 1E-05.


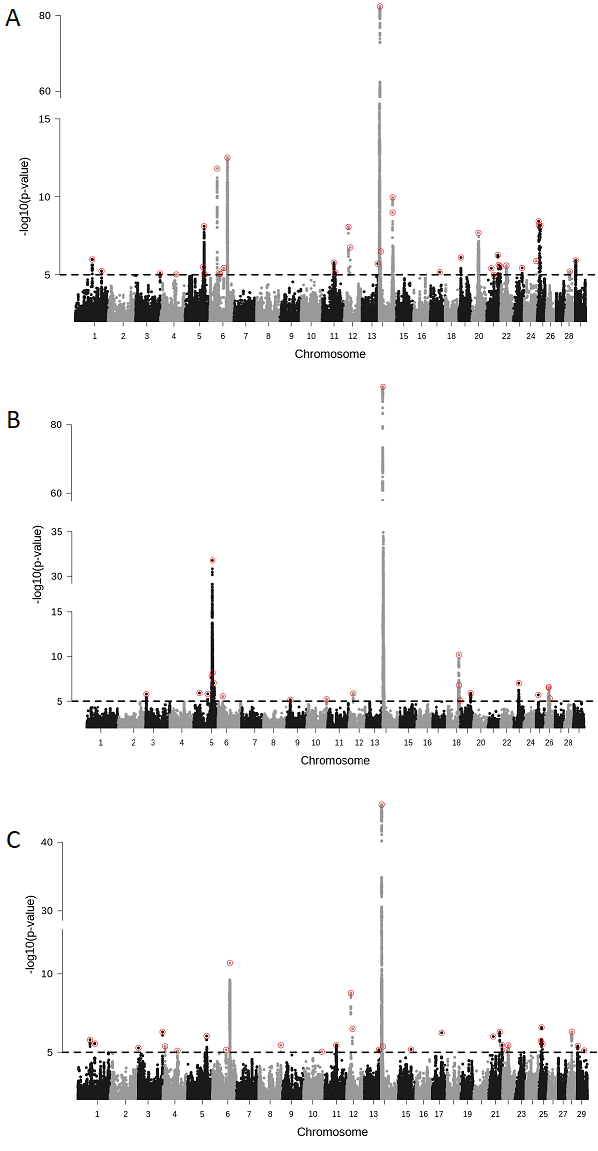


Figure S2. **Manhattan plot of GWAS p-values for 29,107 Holstein cows obtained from 15 million imputed-WGS for**heat tolerance milk (HTMYslope; A), fat (HTFYslope; B), and protein (HTPYslope; C) yield slope traits. The highlighted red points are lead SNPs (most significant) identified using 5 Mb non-overlapping windows at p < 1E-05 (horizontal dashed line).


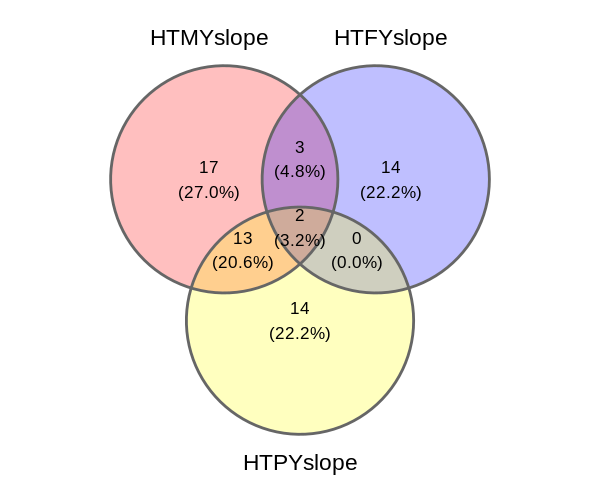


Figure S3. **Overlap of candidate QTLs (p < 1E-05) from single-trait GWAS for heat tolerance milk (HTMYslope), fat (HTFYslope), and protein (HTPYslope) yield slope traits.** QTLs were defined as overlapping if the lead SNPs (most significant) within QTLs are close (within 1 Mb). The two QTLs which overlapped across the 3 slope traits are located around DGAT1 and MGST1 genes.


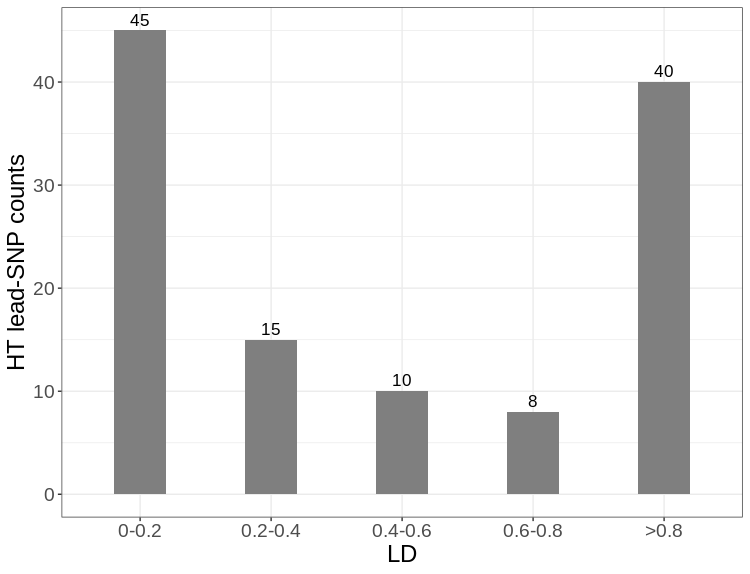


Figure S4. Distribution of linkage disequilibrium (LD) scores between lead SNPs (most significant) for slope traits and nearby (within 1 Mb) lead SNPs for intercept traits identified from single-trait GWAS and multi-trait meta-analyses.


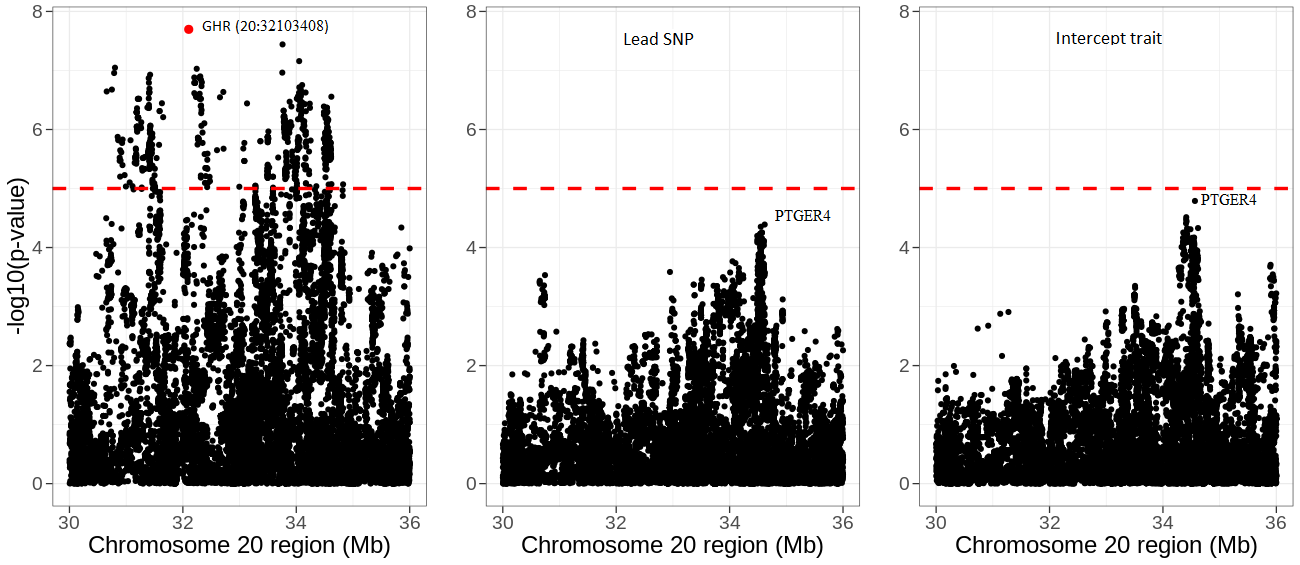


Figure S5. GWAS p-values on chromosome 20 at 30 to 36 Mb for heat tolerance milk yield slope (HTMYslope) trait. The left plot (GWAS p-values before conditional analysis), middle (after conditioning slope on the lead SNP defined as the most significant SNP selected from first-round of GWAS; Chr20:32103408), and right plot (GWAS p-values after conditioning with milk yield intercept trait).


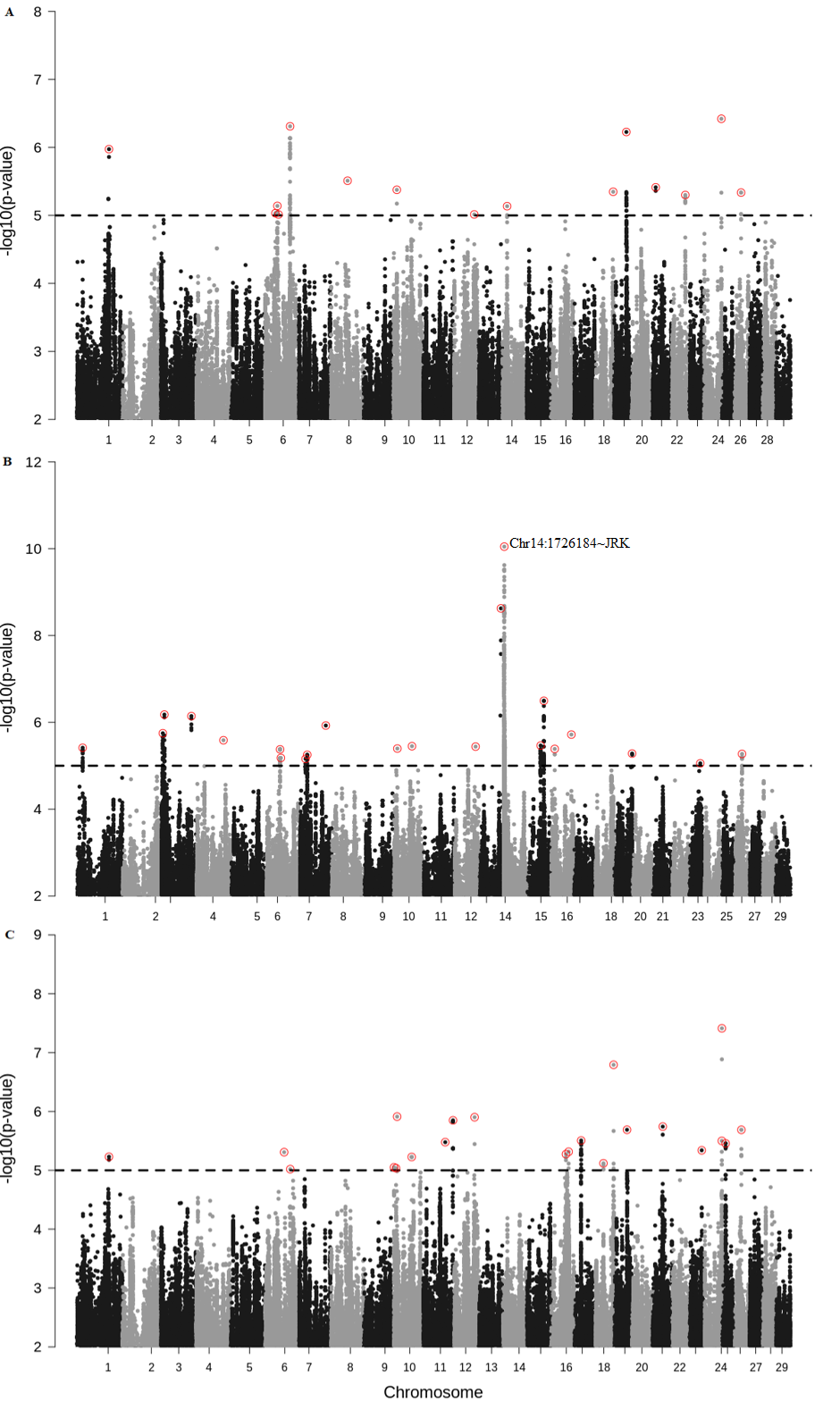


Figure S6. Conditional GWAS results for heat tolerance milk (HTMYslope), fat (HTFYslope), and protein (HTPYslope) yield slope traits. GWAS for A, B and C were conditioned on milk, fat, and protein yield intercept traits, respectively. The highlighted red points represent the lead SNPs (most significant) within 5 Mb non-overlapping windows across the chromosome.


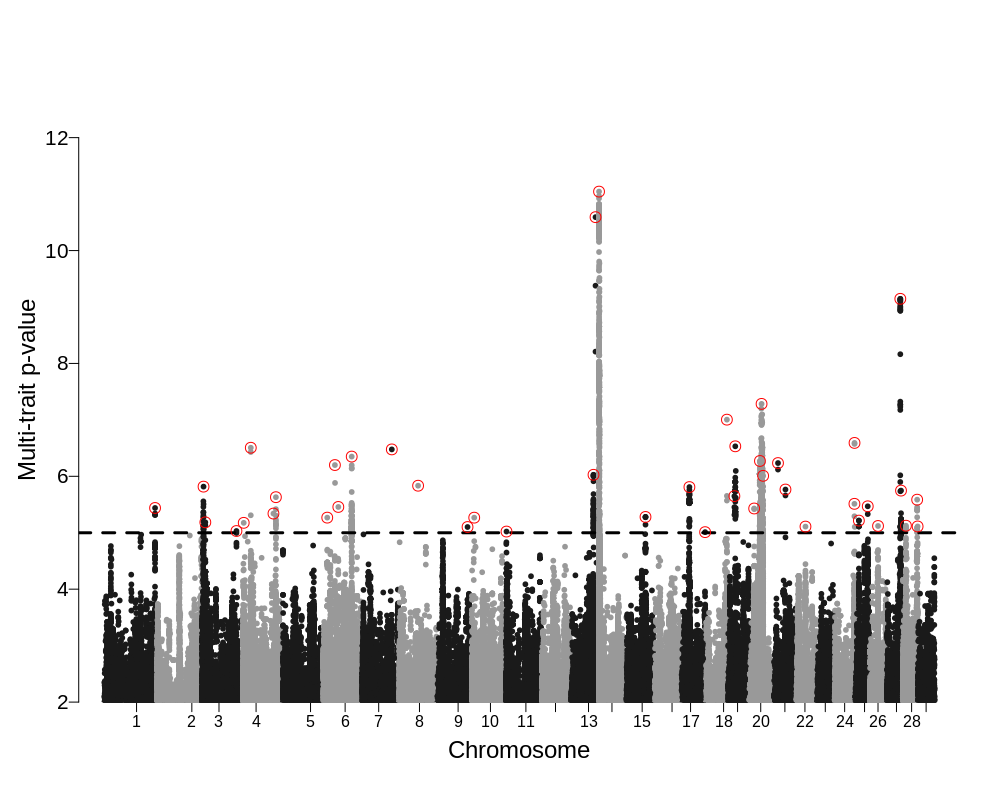
Figure S7. Manhattan plot of p-values obtained from combining conditional single-trait GWAS results for slope traits in the multi-trait meta-analysis. The dashed line is the significant GWAS cut-off at p < 1E-05, while the red circles are the lead SNPs (most significant per QTL).


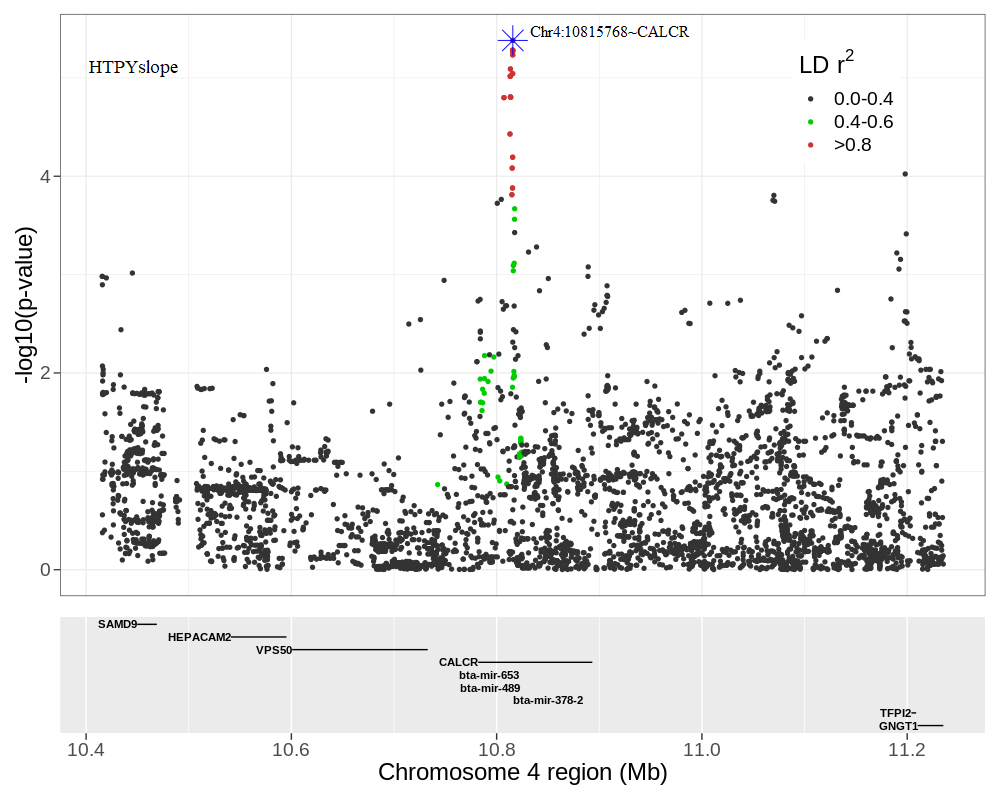


Figure S8. QTL discovery for heat tolerance protein yield slope (HTPYslope) trait around the CALCR gene region in bovine chromosome 4.


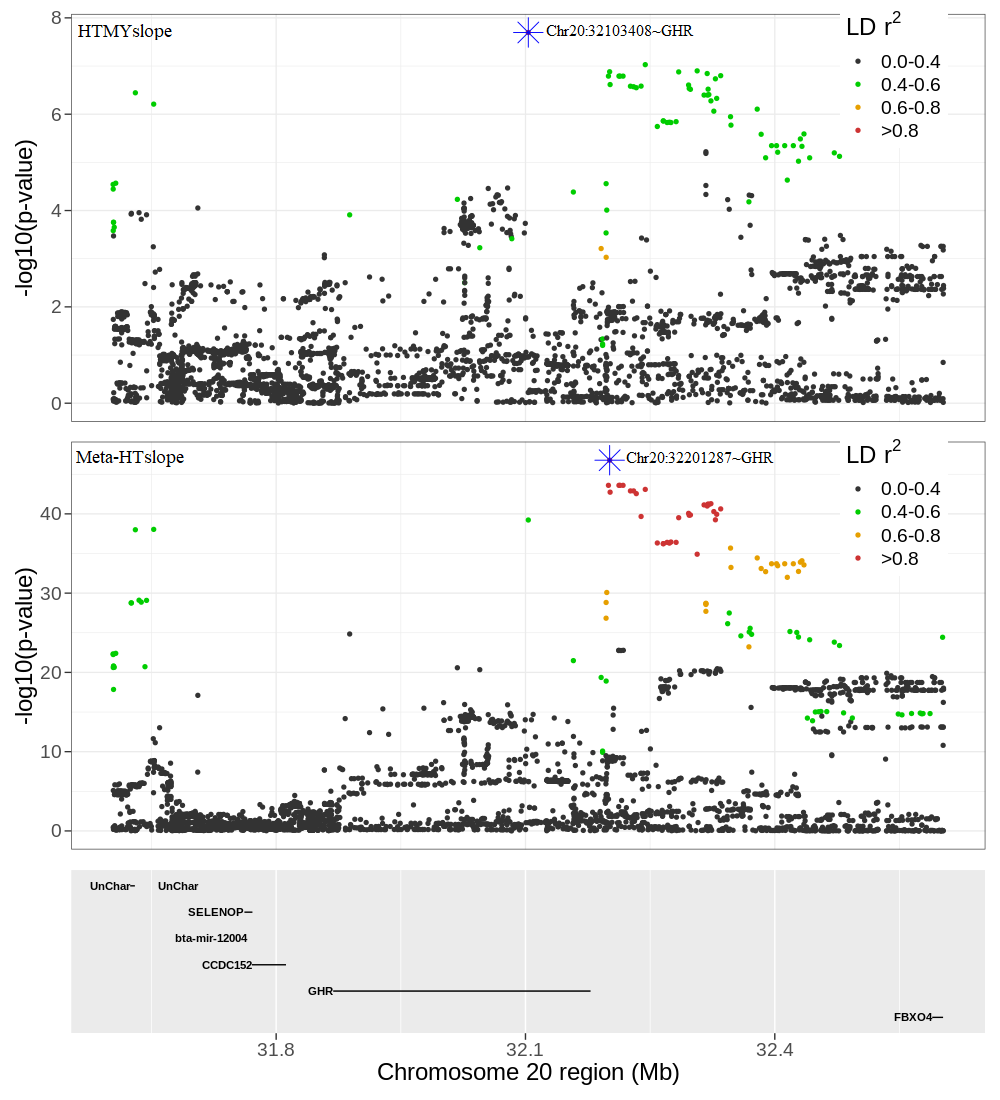


Figure S9. QTL discovery for heat tolerance protein yield slope (HTPYslope) trait and meta-analysis of slope traits (Meta-HTslope) around the GHR gene region in bovine chromosome 20.

## References

1. Cingolani P, Platts A, Wang LL, Coon M, Nguyen T, Wang L, et al. A program for annotating and predicting the effects of single nucleotide polymorphisms, SnpEff: SNPs in the genome of Drosophila melanogaster strain w1118; iso-2; iso-3. Fly. 2012;6(2):80-92.
