# Supplementary material for: New loci and neuronal pathways for resilience to heat stress in cattle
Source: Sci Rep. 2021 Aug 17;11:16619. doi: 10.1038/s41598-021-95816-8 (PMC8371109; doi:10.1038/s41598-021-95816-8)
Supplement: Supplementary file 2 — Supplementary Table S1. [file 41598_2021_95816_MOESM2_ESM.docx]

**Table S1. Number of candidate causal variants (p < 1E-05) for slope traits across different functional classes identified for a) single-trait GWAS, b) meta-analysis of single-trait GWAS results, and c) meta-analysis of conditional single-trait GWAS results of slopes (conditioning each slope trait on the intercept traits).**

|  |  | Candidate causal variants for heat tolerance^1^ | | | | |  |  |
| --- | --- | --- | --- | --- | --- | --- | --- | --- |
| Annotation | All SNPs^2^ | HTMYslope | HTFYslope | HTPYslope | Multi-trait meta-analysis | Conditional analysis | Total^3^ | Enrichment score (p-value)^5^ |
| 3_prime_UTR | 51,358 | 9 | 20 | 7 | 24 | 9 | 31 | 1.23E-07 |
| 5_prime_UTR | 15,076 | 2 | 1 | 1 | 10 | 2 | 12 | 7.15E-05 |
| upstream | 716,960 | 88 | 83 | 77 | 237 | 105 | 277 | 8.54E-25 |
| downstream | 583,346 | 53 | 43 | 46 | 99 | 60 | 127 | 1.67E-01 |
| intronic | 4,574,268 | 254 | 172 | 91 | 552 | 236 | 947 | 8.54E-02 |
| missense | 43,478 | 14 | 13 | 12 | 18 | 14 | 25 | 4.42E-06 |
| synonymous | 54,755 | 21 | 23 | 21 | 29 | 27 | 35 | 4.88E-09 |
| intergenic | 9,035,702 | 762 | 67 | 166 | 559 | 161 | 1,545 | 1.00E+00 |
| Other^4^ | 23,543 | 0 | 0 | 0 | 1 | 0 | 11 | 8.88E-02 |
| Total | 15,098,486 | 1,203 | 422 | 421 | 1,529 | 614 | 3,010 |  |

^1^Defined as lead SNP (most singificant) with an independent QTL plus other significant SNPs in strong LD (r^2^ > 0.8) with the lead SNP, 500 kb up or downstream; ^2^Numbers of variants within each functional class for all the SNPs used in the GWAS; heat tolerance milk (HTMYslope), fat (HTFYslope) and protein (HTPYslope) yield slope traits; ^3^Combined candidate causal variants for all analyses;  ^4^(5_prime_UTR_premature/_start_codon_gain, frameshift, missense&splice, splice_region&intron, stop_gained, etc,); ^5^Calculated from phyper in R v3.61.
