# Supplementary material for: New loci and neuronal pathways for resilience to heat stress in cattle
Source: Sci Rep. 2021 Aug 17;11:16619. doi: 10.1038/s41598-021-95816-8 (PMC8371109; doi:10.1038/s41598-021-95816-8)
Supplement: Supplementary file 6 — Supplementary Table S5. [file 41598_2021_95816_MOESM6_ESM.docx]

**Table S5. Number of variants within different SnpEff predicted impact groups.**

| SnpEff[1] predicted impact group | All SNPs^1^ | Candidate causal varinats^2^ |
| --- | --- | --- |
| Low | 66,704 | 43 |
| Moderate | 44,955 | 26 |
| High | 2,430 | 2 |
| Modifier | 14,984,397 | 2,939 |
| Total | 15,098,486 | 3,010 |

^1^Numbers of variants within each group for all the SNPs used in the GWAS; ^2^Candidate causal variants for heat tolerance (defined as the lead SNP (most significant within an independent QTL) plus other significant SNPs in LD (r^2^ > 0.8) with the lead SNP, 500kb up or downstream) identified from GWAS across all analyses (including variants from the meta-analysis of conditional GWAS results).

# References

1. Cingolani P, Platts A, Wang LL, Coon M, Nguyen T, Wang L, et al. A program for annotating and predicting the effects of single nucleotide polymorphisms, SnpEff: SNPs in the genome of Drosophila melanogaster strain w1118; iso-2; iso-3. Fly. 2012;6(2):80-92.
